# Activation of JNK and IRE1 is critically involved in tanshinone I-induced p62 dependent autophagy in malignant pleural mesothelioma cells: implication of p62 UBA domain

**DOI:** 10.18632/oncotarget.15336

**Published:** 2017-02-15

**Authors:** Jihyun Lee, Eun Jung Sohn, Sangwook Yoon, Gunho Won, Chang Geun Kim, Ji Hoon Jung, Sung-Hoon Kim

**Affiliations:** ^1^ College of Korean Medicine, Kyung Hee University, Dongdaemun-gu, Seoul, 130-701, Republic of Korea

**Keywords:** tanshinone I, autophagy, p62 ^ΔUBA^, IRE1, mesothelioma cells

## Abstract

The aim of present study is to elucidate autophagic mechanism of tanshinone I (Tan I) in H28 and H2452 mesothelioma cells. Herein, Tan I exerted cytotoxicity with autophagic features of autophagy protein 5 (ATG5)/ microtubule-associated protein 1A/1B-light chain 3II (LC3 II) activation, p62/sequestosome 1 (SQSTM1) accumulation and increased number of LC3II punctae, acridine orange-stained cells and autophagic vacuoles. However, 3-methyladenine (3MA) and NH_4_Cl increased cytotoxicity in Tan I treated H28 cells. Furthermore, autophagy flux was enhanced in Tan I-treated H28 cells transfected by RFP-GFP-LC3 constructs, with colocalization of GFP-LC3 punctae with LAMP1 or Lysotracker. Interestingly, C-terminal UBA domain is required for Tan 1 induced aggregation of p62 in H28 cells. Notably, Tan I upregulated CCAAT-enhancer-binding protein homologous protein (CHOP), inositol-requiring protein-1 (IRE1) and p-c-Jun N-terminal kinase (p-JNK), but silencing of IRE1 or p62 and JNK inhibitor SP600125 blocked the LC3II accumulation in Tan I-treated H28 cells. Overall, these findings demonstrate that Tan I exerts antitumor activity through a compromise between apoptosis and p62/SQSTM1-dependent autophagy via activation of JNK and IRE 1 in malignant mesothelioma cells.

## INTRODUCTION

Malignant pleural mesotheliomas are caused by asbestos exposure [1, 2]. Though the recent incidence of malignant mesothelioma has been still increasing due to past exposure to asbestos worldwide, the survival rate of mesothelioma patients is merely 9 to 12 months, due to few effective treatments for mesotheliomas [3].

Autophagy, so called macroautophagy, is a self-digestion process with the features of the formation of double membrane-bound vacuoles called autophagosomes that can be subsequently fused with the lysosome to form the autolysosome [4, 5]. During the autophagosome process, lipid conjugation results in the conversion of the soluble form of LC3 (LC3-I) to the LC3 autophagic vesicle-associated form (LC3-II). Additionally, protein aggregates of p62, also named SQSTM1, elicit direct autophagosome formation [6]. These autophagosomes contain several structural domains, such as the PB1, LIR and UBA domains [7]. Autophagy exhibits dual roles in autophagic survival or type II cell death in several cell types [8, 9]. Tanshinone I (Tan I), Tanshinone IIA, and Cryptotanshinone, the major bioactive compounds of *Salvia miltirrhiza*, have been reported to have anti-inflammatory [10], anti-tumor [11–13], and anti-bacterial effects [14]. Among them, Tan I showed antitumor activities via the inhibition of the growth and invasion of breast [15, 16], prostate [17] and lung [11] cancers. Nevertheless, the underlying autophagic mechanism of Tan I was never investigated in mesothelioma cells until now. Thus, in the current study, an autophagic molecular mechanism of Tan I was elucidated by assessing the essential effect of p62^ΔUBA^ domain andprotein- protein interactions between p62/SQSTM1 and JNK or IRE1 in H28 and H2452 malignant mesothelioma cells.

## RESULTS

### Tan I induces cytotoxicity and autophagy in two mesothelioma cell lines

**Figure 1:**
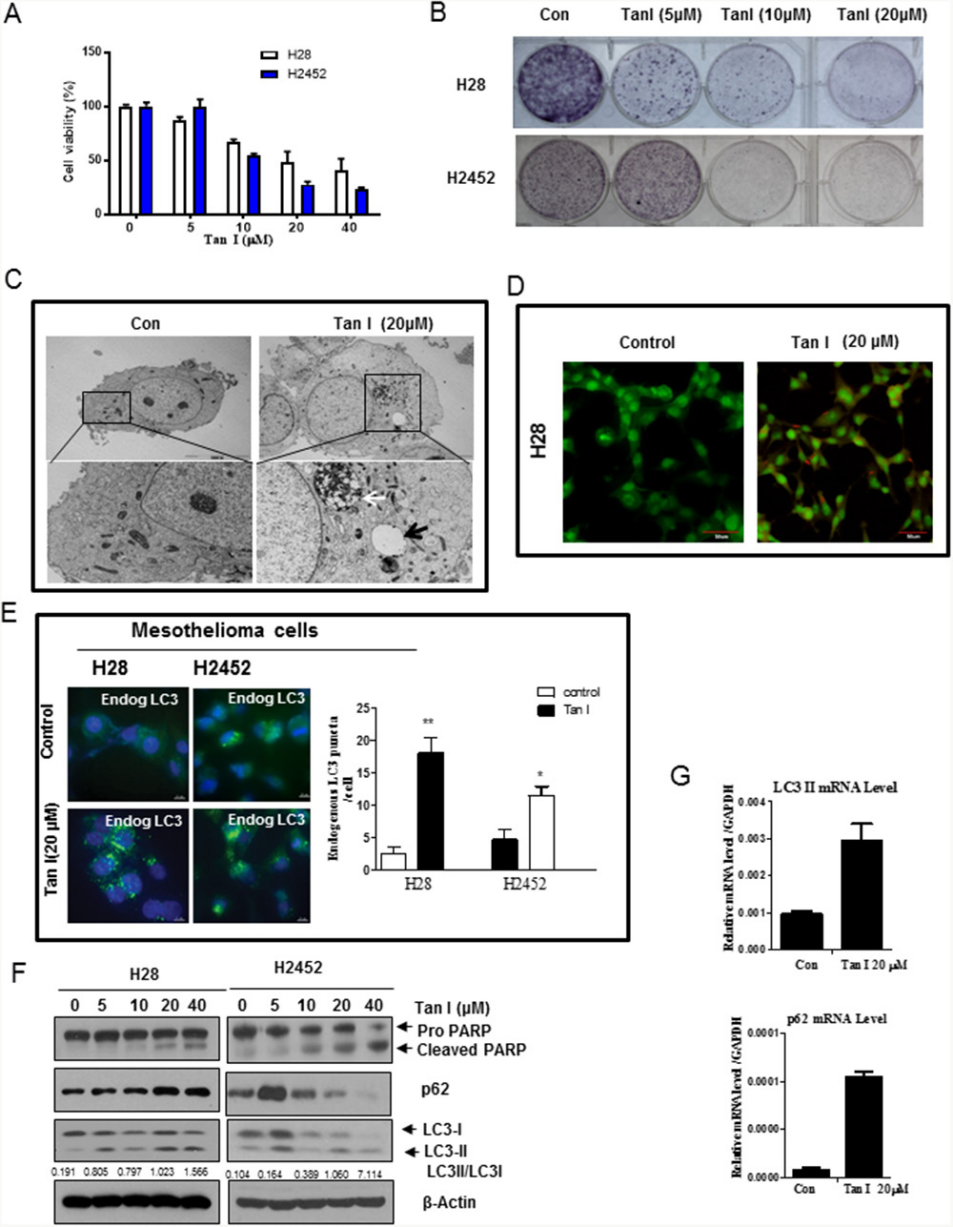
Tan I exerts cytotoxicity and induces autophagy in H28 and H2452 mesothelioma cells **A**. Cytotoxicity of Tan I in the H28 and H2452 mesothelioma cell lines. Two mesothelioma cell lines were treated with various concentrations of Tan I (0, 5, 10, 20, and 40 μM) for 24 h. Cell viability was determined by the MTT assay. **B**. The inhibitory effect of Tan I on colony formation in H28, and H2452 mesothelioma cells. The colony formation assay was performed on mesothelioma cells treated with Tan I (20 μM). **C**. Effect of Tan I on autophagic vacuoles in H28 cells by TEM observation. H28 cells were treated with Tan I (20 μM) for 24 h, and autophagic morphology was observed by TEM. The black arrow indicates an autolysosome that contains the remnants of digested organelles. High-magnification image of the black square (bottom panel). **D**. Effect of Tan I on acidic autophagic vacuoles in Tan I-treated H28 cells. H28 cells were treated with Tan I for 24 h, stained with acridine orange (AO) and observed under the FLUOVIEW FV10i confocal microscopy (Olympus, Tokyo, Japan). **E**. Effect of Tan I (20 μM) on punctae formation of endogenous LC3II in H28 and H2452 cells by immunofluorescence. Bar scale = 10 μM, DAPI-blue, endogenous LC3-Green. **F**. Effect of Tan I on PARP, p62 and LC3II in H28 and H2452 mesothelioma cells. **G**. Effect of Tan I on the mRNA expression of p62 and LC3II in H28 cells by RT-qPCR. Total RNA was isolated from Tan 1 treated H28 cells and RT-PCR was performed as shown in Materials and Methods.

We first examined the cytotoxic effect of Tan I in two mesothelioma cells lines, such as H28 (sarcomatoid) and H2452 (epithelial subtype) cells by MTT assay. Here Tan I induced cytotoxicity (Figure 1A) and increased sub G1 population (Supplementary Figure 1A) in a concentration dependent fashion and reduced colony formation in H28 and H2452 mesothelioma cells (Figure 1B). Consistently, Tan I increased apoptotic portion in H28 and H2452 cells by FACS analysis using Annexin-PI double staining (Supplementary Figure 1B). There are accumulating evidences that autophagy can be a therapeutic target in several cancers [18, 19]. Therefore, we investigated the effect of Tan I on protein expression and the features of autophagy in two mesothelioma cell lines. TEM images revealed that Tan I increased the number of autophagic vacuoles of autophagosomes/autolysosomes with degraded organelles in H28 cells (Figure 1C). To confirm whether Tan I induces late stage autophagy by fusion with lysosomes, H28 cells were stained with acridine orange (AO), which is used for staining acidic vacuoles, including lysosomes, endosomes, and autolysosomes [20, 21], one day after Tan I treatment. As shown in Figure 1D, orange-red color staining with AO dye was observed in Tan I-treated H28 cells, whereas no distinct color change was observed in the untreated control. Furthermore, immunofluorescence showed that Tan I enhanced the formation of LC3 II punctae in two mesothelioma cell lines (Figure 1E). As shown in Figure 1F, Western blotting revealed that Tan I at 20 μM induced the weak cleavage of PARP and also increased the expression of LC3 II in two mesothelioma cells. However, the expression of p62/SQSTM1 was increased in a concentration dependent fashion in Tan I-treated H28 cells, while the expression of p62/SQSTM1 was upregulated at 5 μM of Tan 1, but tended to decrease from 10 μM. Additionally, RT-qPCR analysis revealed that Tan I upregulated p62 and LC3II expression at the mRNA level in H28 cells (Figure 1G), since the p62/SQSTM1 binds to the autophagic effector protein LC3 [22].

### Tan I increases the autophagic flux in H28 cells

**Figure 2:**
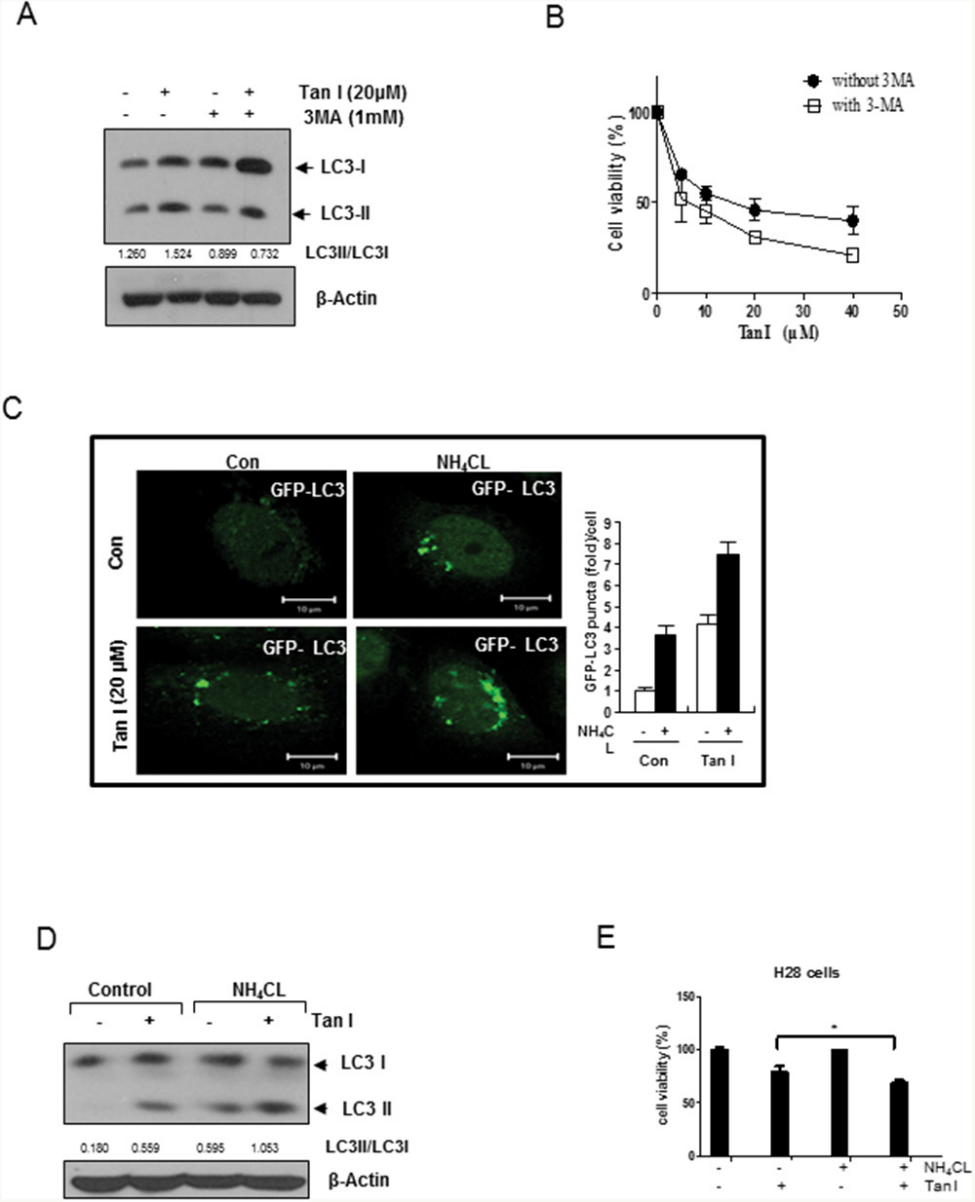
Tan I increases the autophagic flux in H28 cells **A**. Effect of early stage autophagy inhibitor 3 MA on the expression of LC3II in Tan I-treated H28 cells **B**. Effect of 3 MA on cytotoxicity in Tan I-treated H28 cells **C**. Effect of the autolysosome inhibitor NH_4_Cl on GFP-LC3II punctae in Tan I-treated H28 cells. After transient transfection with the GFP-fused LC3 plasmid, H28 cells were treated with Tan I for 24 h and incubated with 3 mM NH_4_Cl for 3 h. The distribution of GFP-LC3II expression was visualized by confocal microscopy. Bar scale=10 μM, GFP-LC3-Green. The bar graph represents the numbers of GFP-LC3II punctae in Tan I-treated H28 cells. The data represent the means ± S.D. of three independent experiments. **D**. Effect of NH_4_Cl on the expression of LC3II in Tan I-treated H28 cells. Western blotting was performed to determine the levels of LC3II in H28 cells treated with Tan I for 24 h. **E**. Effect of NH_4_Cl on the cytotoxicity in Tan I treated H28 cells.

**Figure 3:**
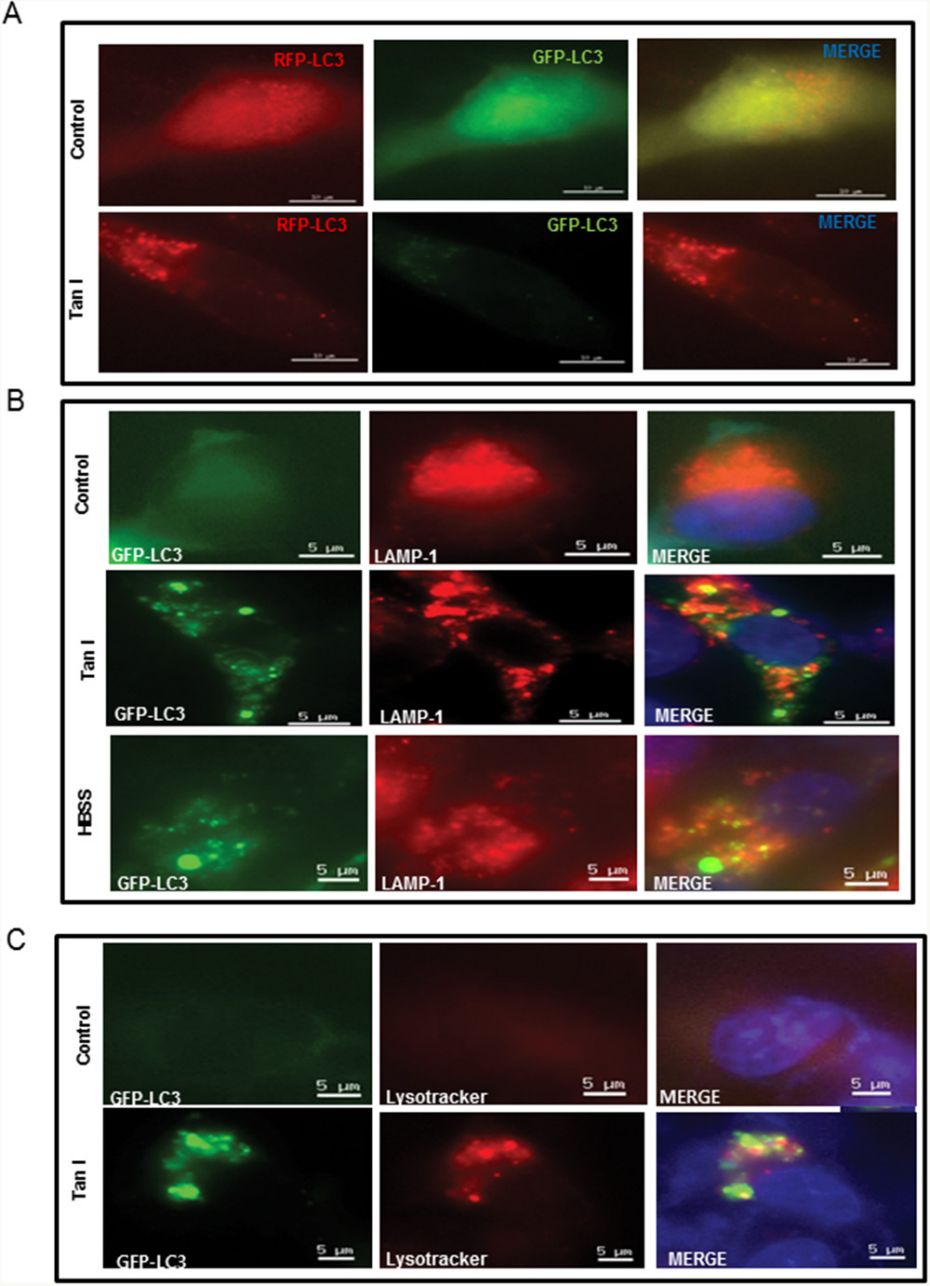
Tan I promotes autolysosome formation through colocalization of GFP-LC3 with LAMP-1 or LysoTracker in H28 cells **A**. Effect of Tan I on autophagy flux in H28 cells. **B**. Effect of Tan I on the colocalization GFP-LC3 and LAMP-1 in H28 cells. The GFP-fused LC3 plasmid was transiently transfected into H28 cells, followed by treatment of Tan I for 24 h. H28 cells were immunostained with an anti-LAMP-1 antibody. The colocalization of GFP-LC3 and LAMP-1 was indicated by the number of yellow spots in the merged images. Bar scale=5 μM, GFP-LC3-Green, DAPI-blue, LAMP1-Red. **C**. Effect of Tan I on the colocalization of GFP-LC3 and LysoTracker in H28 cells. H28 cells were treated with LysoTracker for 30 min, and images were taken under a live cell confocal microscope. The colocalization of GFP-LC3 and LysoTracker was indicated by the number of yellow spots in the merged images. Bar scale =5 μM, GFP-LC3-Green, DAPI-blue, LysoTracker-Red.

To assess whether the elevated levels of LC3 lipidation induced by Tan I in H28 cells were due to fusion with autolysosome or increased degradation, we evaluated the status of the autophagic flux with an early stage autophagy inhibitor 3-MA that inhibits formation of autophagosomes and a late stage autophagy inhibitor NH_4_Cl that blocks fusion with the lysosomes [20]. 3-MA reduced the expression of LC3 II (Figure 2A), but increased cytotoxicity (Figure 2B) in Tan I treated H28 cells. In contrast, after transfection with the GFP-fused LC3II plasmid, the immunofluorescence showed that the number of GFP-LC3II green punctae (Figure 2C), LC3II protein expression (Figure 2D) and cytotoxicity (Figure 2E) by Tan I were increased by NH_4_Cl compared to untreated control (Figure 2C and 2D). To confirm that Tan I induces the autophagy flux, autophagy flux was evaluated by immunofluorescence using LC3 conjugated to red fluorescent protein (RFP) and green fluorescent protein (GFP) (RFP-GFP-LC3) constructs. As shown in Figure 3A, Tan I induced autolysosome (remaining red dots) formation in H28 cells, because the GFP fluorescence was impaired by the acidic lysosomal environment. Furthermore, to investigate whether Tan I generates fusion of autophagosome with the lysosome, the colocation of GFP-LC3II and lysosomal-associated membrane protein 1 (LAMP-1), a marker for endosomal and lysosomal membranes, was assessed in Tan I-treated H28 cells. As shown in Figure 3B, GFP-LC3II punctae were colocalized with LAMP-1 in response to autophagic induction by Tan I. Similarly, punctae of GFP-LC3II induced by Tan I were colocalized with the acetotrophic LysoTracker red DND-99 dye (Figure 3C).

### The p62^ΔUBA^ domain is required in the colocalization of endogenous p62 with GFP-LC3 in Tan I treated H28 cells

**Figure 4:**
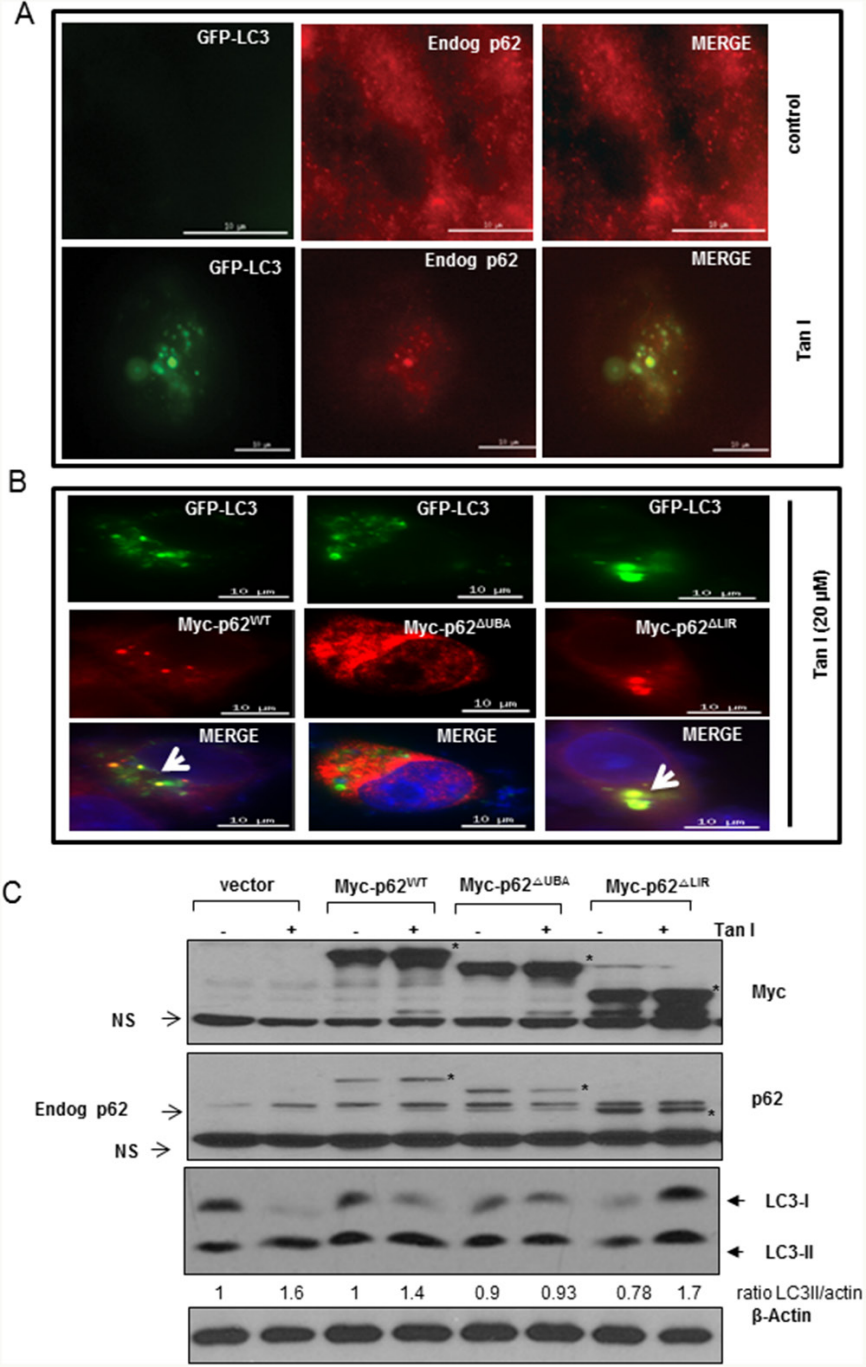
The p62^ΔUBA^ domain is responsible for the colocalization of LC3II and p62 in Tan I treated H28 cells **A**. Effect of Tan I on the colocalization of accumulated p62 with GFP-LC3II in H28 cells. **B**. The p62^ΔUBA^ domain is responsible for LC3II punctae formation by Tan I in H28 cells by immunofluorescence. H28 cells were transiently co-transfected with the Myc-p62^WT^, Myc-p62^ΔUBA^ and Myc-p62^ΔLIR^ domain plasmids. The colocalization of LC3 and aggregates of full-length p62 or its deleted domain p62 plasmids was indicated by arrows. Bar scale =10 μm, GFP-LC3-Green, DAPI-blue, Myc-tagged p62-Red. **C**. Effect of Tan I on the expression of LC3II and p62 in H28 cells transfected with the Myc-p62^WT^, Myc-p62^ΔUBA^ and Myc-p62^ΔLIR^ domain plasmids. Myc-tagged constructs of wild-type (WT) p62, p62^ΔUBA^, and p62^ΔLIR^ were transfected into H28 cells in the presence or absence of Tan I, and Western blotting was performed with antibodies targeting p62, LC3II and β-Actin. An asterisk (*) indicates the p62 expression level of the Myc-tagged p62 constructs.

To investigate the colocalization of LC3II and p62/SQSTM1, H28 cells were with treated with Tan I after GFP-fused LC3II plasmid transfection. As shown in Figure 4A, the immunofluorescence demonstrated that the GFP-fused LC3II punctae, which are autophagosome markers, were colocalized with the p62/SQSTM1 aggregates in GFP-fused LC3II plasmid-transfected H28 cells in the presence of Tan I.

To map which domain of p62/SQSTM1 interacts with LC3II in Tan I-treated H28 cells, Myc-tagged p62/SQSTM1 or GFP-fused LC3II constructs with different deletions were transiently transfected into H28 cells. The colocalization of GFP-fused LC3II and p62/SQSTM1 was analyzed by the Delta Vision imaging system. Our data revealed that full-length p62/SQSTM1 (Myc-p62^wt^) and the LIR-deleted domain mutant (Myc-p62^ΔLIR^) were colocalized with the GFP-fused LC3II punctae, whereas the UBA-deleted domain mutant (Myc-p62^ΔUBA^) was not colocalized with the GFP-fused LC3II punctae (Figure 4B). To investigate whether p62/SQSTM1 interacts with LC3II after transfection with the different Myc tagged-p62/SQSTM1 deletion constructs in H28 cells, their effects on the protein expression of LC3II and p62 were comparatively examined. As shown in Figure 4C, Western blotting also showed that the activation of LC3II and p62 was induced in full-length p62/SQSTM1 or LIR-deleted domain construct-transfected H28 cells, while the expression of p62 was rather decreased and LC3 II conversion was not induced in UBA-deleted domain construct-transfected H28 cells.

### Tan I upregulates IRE1, CHOP and p-JNK in H28 cells

**Figure 5:**
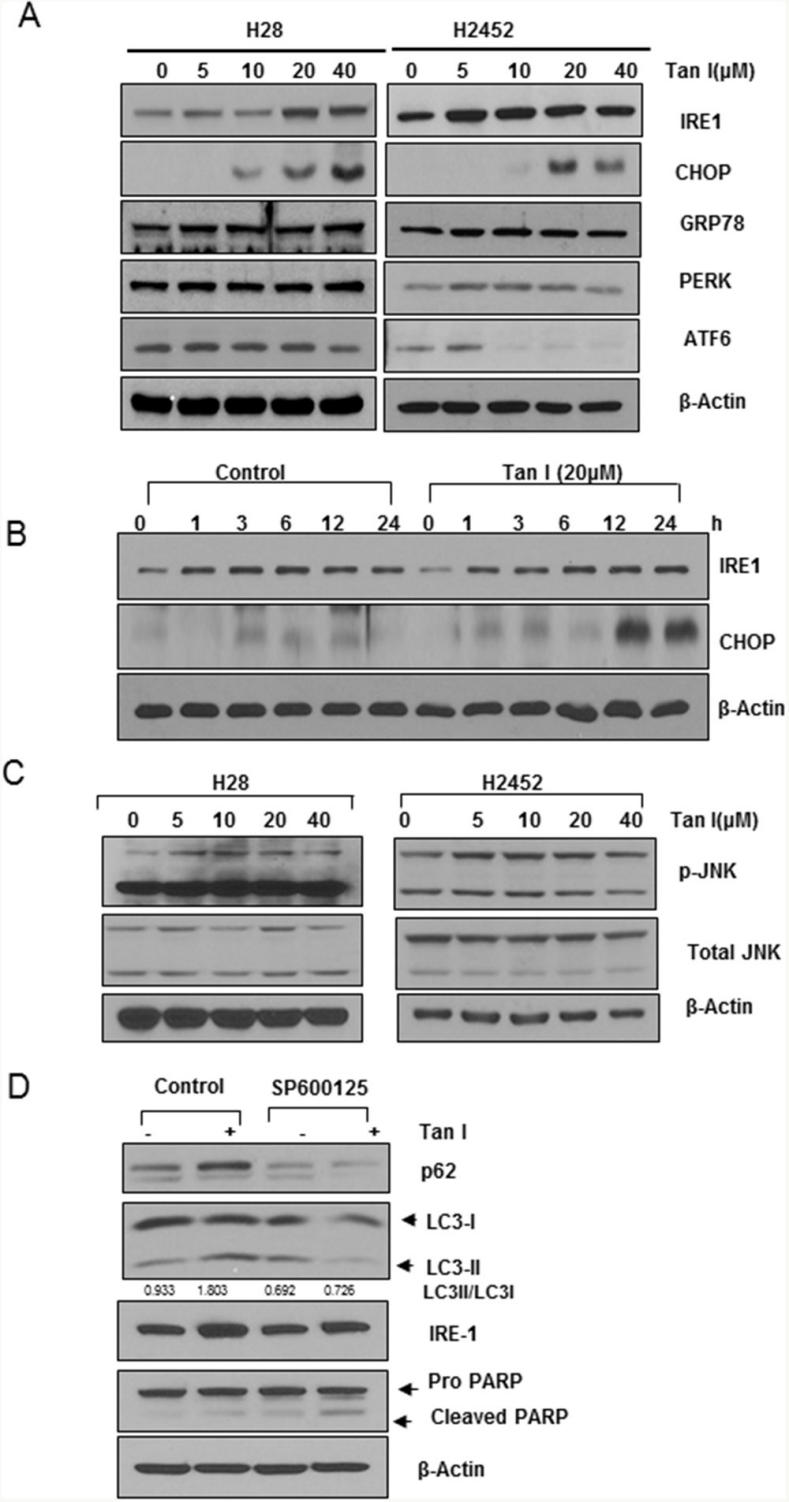
Tan I upregulates IRE1, CHOP and p-JNK in H28 cells **A**. Effect of Tan I on ER stress-related proteins such as GRP78, CHOP, IRE1 and PERK in two mesothelioma cell lines. Western blotting was performed to determine the effect of Tan I on the expression of GRP78, CHOP, PERK, ATF4, ATF6, IRE1 and β-Actin in H28 and H2452 cells. **B**. Time-dependent effect of Tan I on IRE1 and CHOP expression in H28 cells. **C**. Effect of Tan I on the phosphorylation of JNK in H28 and H2452 cells. Two mesothelioma cell lines were exposed to Tan I and Western blotting was performed to determine the effect of Tan I on JNK phosphorylation. **D**. Effect of the JNK inhibitor SP600125 on the expression of p62, LC3II and IRE1 in H28 cells. H28 cells were treated with Tan I (20 μM) in the presence or absence of SP600125 for 24 h. Western blotting was performed to determine the levels of LC3 conversion, and p62/SQSTM1, IRE1 and β-Actin expression.

It was well documented that ER stress is involved in autophagy or apoptosis induction [23, 24]. Thus, the effect of Tan I on ER stress was assessed in two mesothelioma cells. As shown in Figure 5A, Tan I (5, 10, 20 and 40 μM) stimulated the expression of the ER stress markers such as IRE1and CHOP in the two mesothelioma cell lines, but not PERK, GRP78 and ATF6. Consistently, Tan I at 20 μM activated the expression of CHOP and IRE1 in a time-dependent manner in H28 cells (Figure 5B). Furthermore, Tan I increased the phosphorylation of JNK in the H28, and H2452 cells (Figure 5C). However, inhibition of p-JNK by the JNK inhibitor SP600125 suppressed the autophagic ability of Tan I to activate the expression of p62/SQSTM1 and LC3II in H28 cells (Figure 5D).

### Silencing of IRE1 or p62 and also JNK inhibitor SP600125 decrease the activation of LC3 II and p62 in Tan I-treated H28 cells

**Figure 6:**
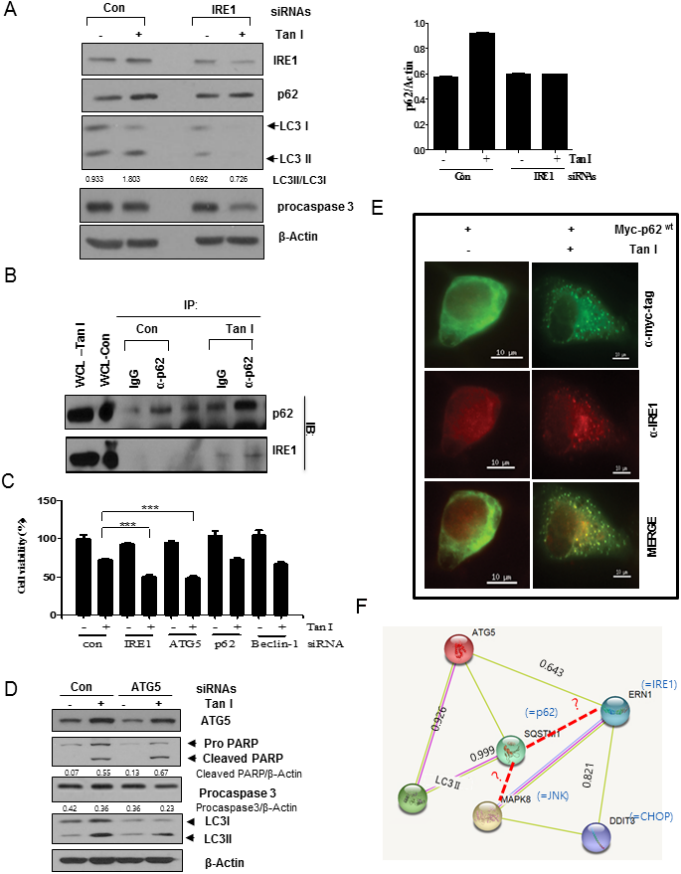
IRE1 and JNK signaling pathways are critically involved in Tan I-induced autophagy in H28 cells **A**. Effect of IRE-1 depletion by siRNA transfection on the ability of Tan I to activate p62 and LC3II in H28 cells. H28 cells were transfected with control or IRE-1 siRNAs. One day after transfection, H28 cells were treated with Tan I and Western blotting was performed to determine the levels of IRE-1, p62/SQSTM1, LC3 and β-Actin. The bar graph represents the densitometric analysis of p62. **B**. Binding of IRE1 and p62 in Tan I-treated H28 cells by the immunoprecipitation (IP) method. H28 cells were treated in the presence or absence of Tan I for 24 h. Endogenous p62/SQSTM1 was immunoprecipitated with a p62 antibody, and Western blotting was performed to determine the binding of IRE1 and p62/SQSTM1. **C**. Depletion of IRE1 or ATG5, but not p62 or Beclin-1, increases cytotoxicity in Tan I treated H28 cells. **D**. Effect of ATG5 depletion by siRNA on PARP, Procaspase 3 and LC3 II by Tan I treatment in H28 cells. **E**. Colocalization of Myc-tagged p62 with endogenous IRE1 was observed by immunofluorescence in Tan I-treated H28 cells. **F**. Protein-protein interactions between ATG5, JNK, IRE1, LC3II and CHOP shown in GeneCards database.

Next, we investigated whether the activation of IRE1 is involved in the p62/SQSTM1 accumulation and LC3II activation in H28 cells. After IRE1 was depleted in H28 cells by the siRNA transfection method, the p62/SQSTM1 and LC3II expression levels were analyzed by Western blotting. As shown in Figure 6A, IRE1 depletion attenuated the expression of p62/SQSTM1 and LC3II to subsequently reduce the expression of procaspase 3 in Tan I-treated H28 cells compared to the untreated control. Notably, IRE1 was pulled down to bind to p62/SQSTM1 by IP Western blotting (Figure 6B) and also p62 was colocalized with endogenous IRE1 following transfection of the Myc-tagged Myc-p62^wt^ construct into Tan I-treated H28 cells by immunofluorescence (Figure 6E). However, silencing of IRE1 or ATG5 increased the cytotoxicity by Tan I, but not depletion of p62 and Beclin 1 in H28 cells (Figure 6C). Consistently, ATG5 depletion reduced LC3 II conversion, and increased PARP cleavage and caspase 3 activation in Tan I treated H28 cells (Figure 6D). Furthermore, our data were supported by protein-protein interactions (PPI) suggested by GeneCards dataset, since PPI binding scores are 0.926 between ATG5 and LC3II, 0.999 between p62/SQSTM1 and LC3II and 0.643 between ATG5 and IRE1 (Figure 6F).

## DISCUSSION

In the current study, the autophagic mechanism of Tan 1 was examined in H28 and H2452 mesothelioma cells to clearly assess the antitumor effect of Tan I. Here, Tan I exerted cytotoxicity and increased sub G1 population in a concentration dependent manner in two mesothelioma cells, but also at a lower concentration 20 μM, increased the expression of LC3II, induced the accumulation of p62/SQSTM1 and formed GFP-fused LC3II punctae in these mesothelioma cells, indicating the autophagic potential of Tan I. Furthermore, autophagic vacuoles of autophagosomes/autolysosomes were observed in Tan I-treated H28 cells by TEM, thereby demonstrating the autophagic effect of Tan I.

The autophagic flux covers autophagosome formation, maturation, fusion with lysosomes, subsequent breakdown and the release of macromolecules back into the cytosol [25, 26]. Here, Tan I increased the autophagic flux by increased acridine orange staining, colocalization of the GFP-LC3II punctae with LysoTracker red or LAMP1 (a late stage marker of autophagic flux) and fluorescent color changes (decreased GFP and maintained RFP) from the tandem RFP-GFP-LC3 constructs in H28 cells, implying complete induction of autophagic flux through the formation of autolysomes by fusion of autophagosomes with lysosomes for degradation in response to Tan I. Conversely, late stage autophagy inhibitor NH_4_Cl increased the accumulation of LC3II punctae, LC3II accumulation and cytotoxicity leading to PARP cleavages in Tan I treated H28 cells. In contrast, treatment with the early stage autophagy inhibitor 3MA also promoted cytotoxicity bur reduced LC3II accumulation in H28 cells, demonstrating that Tan I increases the autophagic flux and that the inhibition of autophagy promotes cytotoxicity in Tan I-treated H28 cells.

The p62/SQSTM1, a selective substrate for autophagy, serves as a scaffold in autophagosomes through several structural domains, including the PB1 (Phox/Bem 1p), TB (TRAF6-binding), LIR (LC3-interacting region) and UBA (ubiquitin-associated) domains [27]. Here, Tan I upregulated the expression of p62/SQSTM1 and LC3II at the protein and mRNA levels, resulting in the colocalization of GFP-LC3 and endogenous p62 in H28 cells, indicating the direct interactions between LC3 and p62. Similarly, resveratrol, PMA and acadesine increased autophagic flux with accumulation of intracellular p62 due to the enhancement of its synthesis as a compensation for autophagic degradation [30–32]. Thus, accumulation of p62/SQSTM1 by Tan 1 can be assumed to be induced via its more active DNA synthesis compared with autophagic degradation.

The p62/SQSTM1 polymerizes via an N-terminal PB1 domain, resulting in the diffuse localization of p62/SQSTM1 [33], interaction with ubiquitinated proteins via the C-terminal UBA domain and also direct binding to LC3 via the LIR motif for the autophagic degradation of p62/SQSTM1 [22, 34, 35]. Here, we found that full-length p62/SQSTM1(Myc-p62^wt^) and the LIR domain-deleted mutant (Myc-p62^ΔLIR^) were colocalized with GFP-fused LC3II punctae, whereas the UBA-deleted domain mutant (Myc-p62^ΔUBA^) was not colocalized with the GFP-fused LC3II punctae. Consistently, the activation of LC3II and p62 was induced in H28 cells transfected with the full-length p62/SQSTM1 and the LIR-deleted domain construct, but not in H28 cells transfected with the UBA-deleted domain construct, demonstrating that the UBA domain of p62/SQSTM1 is required for Tan I-induced autophagy, because the polyubiquitin-binding and homopolymerizing p62 protein aggregates are linked to the autophagic machinery via LC3 conversion [36], implying induction of p62 is the cause rather than effect in Tan I-induced autophagy.

Previous studies have shown that ER stress-activated genes mediated by the unfolded protein response (UPR, PERK, ATF6, and IRE1) are involved in autophagy [37, 38] or apoptosis [39, 40] in several cancers. Furthermore, JNK activation is also involved in autophagy induction in several cancers [41] through close association with ER stress [42]. In the current study, Tan I increased the expression of IRE1, CHOP and p-JNK in a dose- and time-dependent manner in two mesothelioma cells. Interestingly, IRE1 depletion in Tan I-treated H28 cells decreased the expression of p62/SQSTM1 and LC3II through direct binding between IRE1 and p62/SQSTM1 by IP Western blotting and the colocalization of p62 with endogenous IRE1 detected by immunofluorescence, implying the direct interaction of p62/SQSTM1 with IRE1 by Tan I, which was supported by GeneCards dataset. Consistently, silencing of p62 decreased the activated expression of LC3 II, but not IRE1 in Tan I-treated H28 cells, indicating that IRE-1 is an upstream of p62. Furthermore, inhibition of p-JNK by using JNK inhibitor SP600125 decreased the ability of Tan I to activate p62/SQSTM1, LC3II and IRE1 in H28 cells, implying that p-JNK activates the signaling of p62 or IRE1 through the autophagy process, which was supported by the previous report that resveratrol-mediated JNK activation increased the expression of p62/SQSTM1 protein and mRNA levels [30].

Although Tan I can be postulated to exhibit antitumor activity in mesothelioma cells through the compromise between apoptosis at higher concentrations and autophagy at lower concentrations, further studies are required to elucidate the underlying molecular mechanisms of Tan I in autophagy and apoptosis through animal study and more mechanistic work in the future. Taken together, these findings demonstrate that the activation of JNK and IRE1 is critically involved in Tan I-induced p62/SQSTM1-dependent autophagy in malignant pleural mesothelioma cells.

## MATERIALS AND METHODS

### Chemicals

Tanshinone I (T5330) and NH_4_CL (A9434) were purchased from Sigma. The JNK inhibitor SP600125 and 3MA were obtained from CALBIOCHEM (LL20119) and Santa Cruz (5142-23-4), respectively.

### Cell culture

The mesothelioma cell lines H28 (ATCC® CRL 5820™), and H2452 (ATCC^®^ CRL-5946^™^) were purchased from the American Type Culture Collection (ATCC). The cells were cultured at 37 °C in a humidified atmosphere of 95% air and 5% CO_2_ with RPMI 1640 medium (Invitrogen, LM011-01) supplemented with 10% fetal bovine serum (FBS) (Welgene, S001-07) and antibiotic-antimycotic solution (Invitrogen, LS203-01).

### Cytotoxicity assay

To examine the cytotoxicity of Tan I in H28 and H2452 mesothelioma cells, MTT assay was conducted. The cells at a density of 2 × 10^4^ grown to 80% confluency in 96-well plates were exposed to various concentrations of Tan I for 1–2 days and also incubated with an MTT working solution (5 mg/ml in PBS) at 37 °C for 2 h. The optical density (OD) was measured at 570 nm using a microplate reader (Sunrise, TECAN). Cell viability was calculated as the percentage of viable cells versus untreated cells by the following equation: Cell viability (%) = [OD (Tan I) – OD (Blank)] / [OD (untreated) – OD (Blank)] × 100.

### Detection of acidic vesicular organelles

Acridine orange staining (Sigma Chemical Co, A6014) was performed to identify the formation of acidic vesicular organelles (AVOs). H28 cells were stained with 1 μg/ml acridine orange with or without Tan 1 for 15 min and observed under a FLUOVIEW FV10i confocal microscope (Olympus).

### Transmission electron microscopy (TEM) observation

The autophagic features were observed in Tan I-treated H28 cells by using TEM. Briefly, H28 cells were pre-fixed with Karnovsky’s fixative (2% glutaraldehyde, 2% paraformaldehyde, and 0.1 M phosphate buffer) for 6h. After washing with 0.1 M PBS, the cells were postfixed with 2 % OsO_4_ with 0.1 M phosphate buffer for 2 h. The cells were washed twice in PBS, followed by dehydration with a graded ethanol series and infiltration with propylene oxide for 10 min. Then, the cells were embedded with an EPON mixture (EPON 812, MNA, DDSA, and DMP30) and propylene oxide. Ultrathin sections were stained with uranyl acetate and lead citrate and observed using an EM208S transmission electron microscope (Philips).

### Autophagic flux assay and autophagy inhibitor study

To investigate whether autophagosome accumulation in response to increased LC3-II punctae was induced directly by enhanced autophagosome formation or lysosomal degradation, autophagy flux assay was performed. Tandem sensor RFP-GFP-LC3 constructs (a gift from Prof. Hongbo Hu from the Department of Nutrition and Health, College of Food Science and Nutritional Engineering, China Agricultural University) were transfected into H28 cells by a transient transfection method using Lipofectamine 2000 (Invitrogen) and exposed to 20 μM Tan I for 24 h. Then, the red and green channel signals were observed in Tan 1-treated H28 cells under the Delta Vision imaging system (Applied Precision). Additionally, Tan I (20 μM)-treated cells were co-incubated with 3 mM NH_4_Cl(Sigma) and 1 mM 3MA (CALBIOCHEM) for 3 h and observed under a FLUOVIEW FV10i confocal microscope (Olympus). Also MTT assay was conducted in H28 cells after Tan 1 treatment for 24 h with or without 3 mM NH_4_Cl (Sigma) and 1 mM 3MA (CALBIOCHEM).

### LysoTracker and immunostaining

One day after transfection of GFP-LC3 plasmid into H28 cells, the cells were treated with Tan I for 24 h, stained with 50 nm LAMP-1 or LysoTracker red (Invitrogen, L-7528) for 30 min, and then washed three times with PBS. The cells were fixed with 4% paraformaldehyde in PBS for 20 min and permeabilized with 1% Triton-X 100 in PBS for 5 min. The fixed cells were incubated with an anti-LC3II antibody (Cell signaling, 3868S) in 1% BSA-PBS overnight at 4 °C. The fixed cells were washed and then stained with the corresponding Alexa Fluor fluorescent antibody (Life Technologies, A-11008) for 30 min at RT. The cell nuclei were counterstained with 1 μg/ml DAPI (Sigma, ZA0629), and the cells were mounted onto slides. The images were obtained using the Delta Vision imaging system (Applied Precision).

### GFP–LC3II–p62 domain plasmid transfection

H28 cells were transfected with 1 μg of the GFP-fused LC3 II expression plasmid and/or Myc tagged-p62/SQSTM1 deletion plasmids (a gift from prof. Jorge Moscat, Sanford Burnham Prebys Medical Discovery Institute, USA) using the Fugene 6 (Applied Biosystems, 11815091001) transfection reagent. One day later, the transfected cells were treated with Tan 1 for 24 h, and the distribution of GFP-LC3II was visualized with the Delta Vision imaging system (Applied Precision).

### Real-time quantitative RT-PCR analysis (RT-qPCR)

Total RNA was isolated from Tan 1 treated H28 cells by using the RNeasy mini kit (Qiagen, Valencia, CA) according to the manufacturer’s instructions and reverse transcribed using M-MLV reverse transcriptase (Promega, Madison, WI). The cDNA was amplified by PCR using the synthesized specific primers (Bioneer, Daejeon, Korea):

**Table:** 

p62/ SQSTM1	Forward	5′-ATC GGA GGA TCC GAG TGT-3′
	Reverse	5′-TGG CTG TGA GCT GCT CTT-3′
LC3II	Forward	5′-GATGTCCGACTTATTCGAGAGC -3′
	Reverse	5′-TTGAGCTGTAAGCGCCTTCTA-3′
GAPDH	Forward	5′-CTGCACCACCAACTGCTTAG-3′
	Reverse	5′-AGGTCCACCACTGACACGTT-3′

An equal amount of cDNA was amplified by PCR using ExTaq DNA polymerase (Takara Bio Inc, Shiga, Japan) and separated on1-2% agarose gel. RT-qPCR was operated with the light cycler TM instrument (Roche Applied Sciences, Indianapolis, IN) according to the manufacturer’s protocol. The mRNA level of GAPDH was used to normalize the expression of genes of interest.

### Western blotting

Total proteins were extracted from Tan I-treated H28 cells using lysis buffer (Institute de Biologie Structurale-BR002) (50 mM Tris–HCl, pH 7.4, 150 mM NaCl, 1% Triton X-100, 0.1% SDS, 1 mM EDTA, 1 mM Na3VO4, 1 mM NaF, and protease inhibitor cocktail). The proteins were separated on 10–12.5% SDS-PAGE gels and transferred to Hybond ECL transfer nitrocellulose membranes (GE Healthcare Bio-Science). After blocking with 5% nonfat dry milk, the membranes were incubated with the desired primary antibodies [p62 (5114S), LC3II (3868S), poly(ADP)-ribose polymerase (PARP) (12061), CHOP (2898S), PERK (12185), ATF4 (11815S), ATG5 (FL-25), Beclin-1 (4445), IRE1 (3294), β-Actin (12262) (Cell signaling), and ATF6 (Santa Cruz Biotechnology, sc22799)], followed by a horseradish peroxidase-labeled anti-rabbit (sc2313) or mouse IgG (sc10198). The immune-reactive bands were visualized with an enhanced chemiluminescence detection kit (Amersham Pharmacia).

### Immunoprecipitation/ immunoblotting (IP-western blotting)

For the immunoprecipitation (IP) experiment, H28 cells were treated with or without Tan I (20 μM) for 24 h. The cells were lysed for 30 min on ice with lysis buffer containing a cocktail of protease/phosphatase inhibitors (Roche, 10711400). The lysates were pre-cleared with A/G agarose bead (Santa Cruz Biotechnology, sc-2003) and then immunoprecipitated at 4 °C with antibodies directed against p62 (Cell signaling, 7695) and rabbit IgG (Santa Cruz biotechnology, sc2313). The IP-Western blotting was performed to determine the binding expression levels between p62 and IRE1.

### RNA interference assay

Scrambled controls and p62 and IRE1 small interfering RNA (siRNA) were obtained from Invitrogen (169688). IRE1 siRNAs were obtained from Bioneer. Transfection with these siRNA plasmids was performed using the Interferin^TM^ transfection reagent (Polyplus-transfection Inc., 11062400) according to the protocol provided by the manufacturer.

### Statistical analysis

All experiments were performed at least three times. The data were expressed as the means ± S.D. Statistical significance between groups was evaluated by Student’s *t*-test using SigmaPlot software (Systat Software Inc., USA).

## SUPPLEMENTARY FIGURE



## Abbreviations

Tan I: Tanshinone I
3-MA: 3-methyladenine
AO: acridine orange
ATG5: autophagy protein 5
CHOP: CCAAT/enhancer-binding protein homologous protein
SQSTM1: sequestosome 1; endoplasmic reticulum
GRP78: glucose-regulated protein 78
IRE1: inositol-requiring kinase 1
PERK: protein kinase RNA-like ER kinase
ATF6: activating transcription factor6
JNK: c-Jun N-terminal kinase
LAMP-1: lysosomal-associated membrane protein 1
LC3: microtubule-associated protein light chain 3
MTT: 3-(4, 5-dimethylthiazol-2-yl)-2,5-diphenyltetrazolium bromide
LIR: LC3-interacting region
UBA: ubiquitin-associated
NH4Cl: ammonium chloride
TB: TRAF6-binding
PB1: Phox/Bem 1p
UBA: ubiquitin-associated
UPR: unfolded protein response
TEM: transmission electron microscopy
PMA: phorbol 12 myristate 13 acetate
PBS: phosphate buffered saline
DAPI: 4′,6-diamidino-2-phenylindole
SDS: sodium dodecyl sulfate (SDS)
EDTA: ethylenediaminetetraacetic acid

## References

[1] Robinson BW, Lake RA (2005). Advances in malignant mesothelioma. N Engl J Med.

[2] Zucali PA, Ceresoli GL, De Vincenzo F, Simonelli M, Lorenzi E, Gianoncelli L, Santoro A (2011). Advances in the biology of malignant pleural mesothelioma. Cancer Treat Rev.

[3] Stahel RA, Weder W (2009). Improving the outcome in malignant pleural mesothelioma: nonaggressive or aggressive approach?. Curr Opin Oncol.

[4] Salazar M, Hernandez-Tiedra S, Torres S, Lorente M, Guzman M, Velasco G (2011). Detecting autophagy in response to ER stress signals in cancer. Methods in enzymology.

[5] He C, Klionsky DJ (2009). Regulation mechanisms and signaling pathways of autophagy. Annu Rev Genet.

[6] Itakura E, Mizushima N (2011). p62 Targeting to the autophagosome formation site requires self-oligomerization but not LC3 binding. The Journal of cell biology.

[7] Seibenhener ML, Geetha T, Wooten MW (2007). Sequestosome 1/p62--more than just a scaffold. FEBS letters.

[8] Codogno P, Meijer AJ (2005). Autophagy and signaling: their role in cell survival and cell death. Cell death and differentiation.

[9] Yonekawa T, Thorburn A (2013). Autophagy and cell death. Essays Biochem.

[10] Zheng S, Ren Z, Zhang Y, Qiao Y (2014). Anti-inflammatory mechanism research of tanshinone II A by module-based network analysis. Biomed Mater Eng.

[11] Lee CY, Sher HF, Chen HW, Liu CC, Chen CH, Lin CS, Yang PC, Tsay HS, Chen JJ (2008). Anticancer effects of tanshinone I in human non-small cell lung cancer. Mol Cancer Ther.

[12] Li Y, Gong Y, Li L, Abdolmaleky HM, Zhou JR (2013). Bioactive tanshinone I inhibits the growth of lung cancer in part via downregulation of Aurora A function. Molecular carcinogenesis.

[13] Wang X, Morris-Natschke SL, Lee KH (2007). New developments in the chemistry and biology of the bioactive constituents of Tanshen. Med Res Rev.

[14] Lee DS, Lee SH, Noh JG, Hong SD (1999). Antibacterial activities of cryptotanshinone and dihydrotanshinone I from a medicinal herb, Salvia miltiorrhiza Bunge. Bioscience, biotechnology, and biochemistry.

[15] Nizamutdinova IT, Lee GW, Son KH, Jeon SJ, Kang SS, Kim YS, Lee JH, Seo HG, Chang KC, Kim HJ (2008). Tanshinone I effectively induces apoptosis in estrogen receptor-positive (MCF-7) and estrogen receptor-negative (MDA-MB-231) breast cancer cells. International journal of oncology.

[16] Nizamutdinova IT, Lee GW, Lee JS, Cho MK, Son KH, Jeon SJ, Kang SS, Kim YS, Lee JH, Seo HG, Chang KC, Kim HJ (2008). Tanshinone I suppresses growth and invasion of human breast cancer cells, MDA-MB-231, through regulation of adhesion molecules. Carcinogenesis.

[17] Zhang Y, Won SH, Jiang C, Lee HJ, Jeong SJ, Lee EO, Zhang J, Ye M, Kim SH, Lu J (2012). Tanshinones from Chinese medicinal herb Danshen (Salvia miltiorrhiza Bunge) suppress prostate cancer growth and androgen receptor signaling. Pharmaceutical research.

[18] Suman S, Das TP, Reddy R, Nyakeriga AM, Luevano JE, Konwar D, Pahari P, Damodaran C (2014). The pro-apoptotic role of autophagy in breast cancer. British journal of cancer.

[19] Sharma K, Le N, Alotaibi M, Gewirtz DA (2014). Cytotoxic autophagy in cancer therapy. International journal of molecular sciences.

[20] Mizushima N, Yoshimori T, Levine B (2010). Methods in mammalian autophagy research. Cell.

[21] Pierzynska-Mach A, Janowski PA, Dobrucki JW (2014). Evaluation of acridine orange, LysoTracker Red, and quinacrine as fluorescent probes for long-term tracking of acidic vesicles. Cytometry Part A.

[22] Pankiv S, Clausen TH, Lamark T, Brech A, Bruun JA, Outzen H, Overvatn A, Bjorkoy G, Johansen T (2007). p62/SQSTM1 binds directly to Atg8/LC3 to facilitate degradation of ubiquitinated protein aggregates by autophagy. The Journal of biological chemistry.

[23] Hayashi-Nishino M, Fujita N, Noda T, Yamaguchi A, Yoshimori T, Yamamoto A (2009). A subdomain of the endoplasmic reticulum forms a cradle for autophagosome formation. Nature cell biology.

[24] Kim MK, Park GH, Eo HJ, Song HM, Lee JW, Kwon MJ, Koo JS, Jeong JB (2015). Tanshinone I induces cyclin D1 proteasomal degradation in an ERK1/2 dependent way in human colorectal cancer cells. Fitoterapia.

[25] Zhang XJ, Chen S, Huang KX, Le WD (2013). Why should autophagic flux be assessed?. Acta pharmacologica Sinica.

[26] Singh K, Sharma A, Mir MC, Drazba JA, Heston WD, Magi-Galluzzi C, Hansel D, Rubin BP, Klein EA, Almasan A (2014). Autophagic flux determines cell death and survival in response to Apo2L/TRAIL (dulanermin). Molecular cancer.

[27] Moscat J, Diaz-Meco MT (2009). p62 at the crossroads of autophagy, apoptosis, and cancer. Cell.

[28] Puissant A, Fenouille N, Auberger P (2012). When autophagy meets cancer through p62/SQSTM1. American journal of cancer research.

[29] Jin Z, Li Y, Pitti R, Lawrence D, Pham VC, Lill JR, Ashkenazi A (2009). Cullin3-based polyubiquitination and p62-dependent aggregation of caspase-8 mediate extrinsic apoptosis signaling. Cell.

[30] Puissant A, Robert G, Fenouille N, Luciano F, Cassuto JP, Raynaud S, Auberger P (2010). Resveratrol promotes autophagic cell death in chronic myelogenous leukemia cells via JNK-mediated p62/SQSTM1 expression and AMPK activation. Cancer Res.

[31] Robert G, Ben Sahra I, Puissant A, Colosetti P, Belhacene N, Gounon P, Hofman P, Bost F, Cassuto JP, Auberger P (2009). Acadesine kills chronic myelogenous leukemia (CML) cells through PKC-dependent induction of autophagic cell death. PloS one.

[32] Colosetti P, Puissant A, Robert G, Luciano F, Jauel A, Gounon P, Cassuto JP, Auberger P (2009). Autophagy is an important event for megakaryocytic differentiation of the chronic myelogenous leukemia K562 cell line. Autophagy.

[33] Lamark T, Perander M, Outzen H, Kristiansen K, Overvatn A, Michaelsen E, Bjorkoy G, Johansen T (2003). Interaction codes within the family of mammalian Phox and Bem1p domain-containing proteins. The Journal of biological chemistry.

[34] Ichimura Y, Kumanomidou T, Sou YS, Mizushima T, Ezaki J, Ueno T, Kominami E, Yamane T, Tanaka K, Komatsu M (2008). Structural basis for sorting mechanism of p62 in selective autophagy. The Journal of biological chemistry.

[35] Bjorkoy G, Lamark T, Pankiv S, Overvatn A, Brech A, Johansen T (2009). Monitoring autophagic degradation of p62/SQSTM1. Methods in enzymology.

[36] Heinen C, Garner TP, Long J, Bottcher C, Ralston SH, Cavey JR, Searle MS, Layfield R, Dantuma NP (2010). Mutant p62/SQSTM1 UBA domains linked to Paget’s disease of bone differ in their abilities to function as stabilization signals. FEBS letters.

[37] Deegan S, Koryga I, Glynn SA, Gupta S, Gorman AM, Samali A (2015). A close connection between the PERK and IRE arms of the UPR and the transcriptional regulation of autophagy. Biochemical and biophysical research communications.

[38] Zhou Y, Liang X, Chang H, Shu F, Wu Y, Zhang T, Fu Y, Zhang Q, Zhu JD, Mi M (2014). Ampelopsin-induced autophagy protects breast cancer cells from apoptosis through Akt-mTOR pathway via endoplasmic reticulum stress. Cancer science.

[39] Wu XY, Fan RT, Yan XH, Cui J, Xu JL, Gu H, Gao YJ (2015). Endoplasmic reticulum stress protects human thyroid carcinoma cell lines against ionizing radiation-induced apoptosis. Molecular medicine reports.

[40] Min KJ, Jung KJ, Kwon TK (2014). Carnosic Acid Induces Apoptosis Through Reactive Oxygen Species-mediated Endoplasmic Reticulum Stress Induction in Human Renal Carcinoma Caki Cells. Journal of cancer prevention.

[41] Zhou H, Shen T, Shang C, Luo Y, Liu L, Yan J, Li Y, Huang S (2014). Ciclopirox induces autophagy through reactive oxygen species-mediated activation of JNK signaling pathway. Oncotarget.

[42] Cheng X, Liu H, Jiang CC, Fang L, Chen C, Zhang XD, Jiang ZW (2014). Connecting endoplasmic reticulum stress to autophagy through IRE1/JNK/beclin-1 in breast cancer cells. International journal of molecular medicine.

