# Supplementary material for: Activation of JNK and IRE1 is critically involved in tanshinone I-induced p62 dependent autophagy in malignant pleural mesothelioma cells: implication of p62 UBA domain
Source: Oncotarget. 2017 Feb 15;8(15):25032–45. doi: 10.18632/oncotarget.15336 (PMC5421907; doi:10.18632/oncotarget.15336)
Supplement: Supplementary file 1 [file oncotarget-08-25032-s001.pdf]

Activation of JNK and IRE1 is critically involved in tanshinone I-induced p62 dependent autophagy in malignant pleural mesothelioma cells: implication of p62 UBA domain

SUPPLEMENTARY MATERIALS AND METHODS

Cell cycle analysis

Tan I treated mesothelioma cells (H2452, and H28) were fixed in 75% ethanol at -20˚C and were resuspended in PBS containing RNase A (1 mg/ml). Following incubation for 1 h at 37˚C, the fixed cells were stained with propidium iodide (50 μg/ml) for 30 min at room temperature in dark. To analyze the DNA contents of the stained cells, CellQuest Software with the FACSCalibur flow cytometry (Becton Dickinson, Franklin Lakes, NJ) was used.

Apoptosis detection by flow cytometry

Apoptosis detection was conducted by Flow cytometric analysis. H28 and H2452 cells were stained using Annexin V and PI antibodies. Stained cells were analyzed with a FACSCalibur flow cytometer.

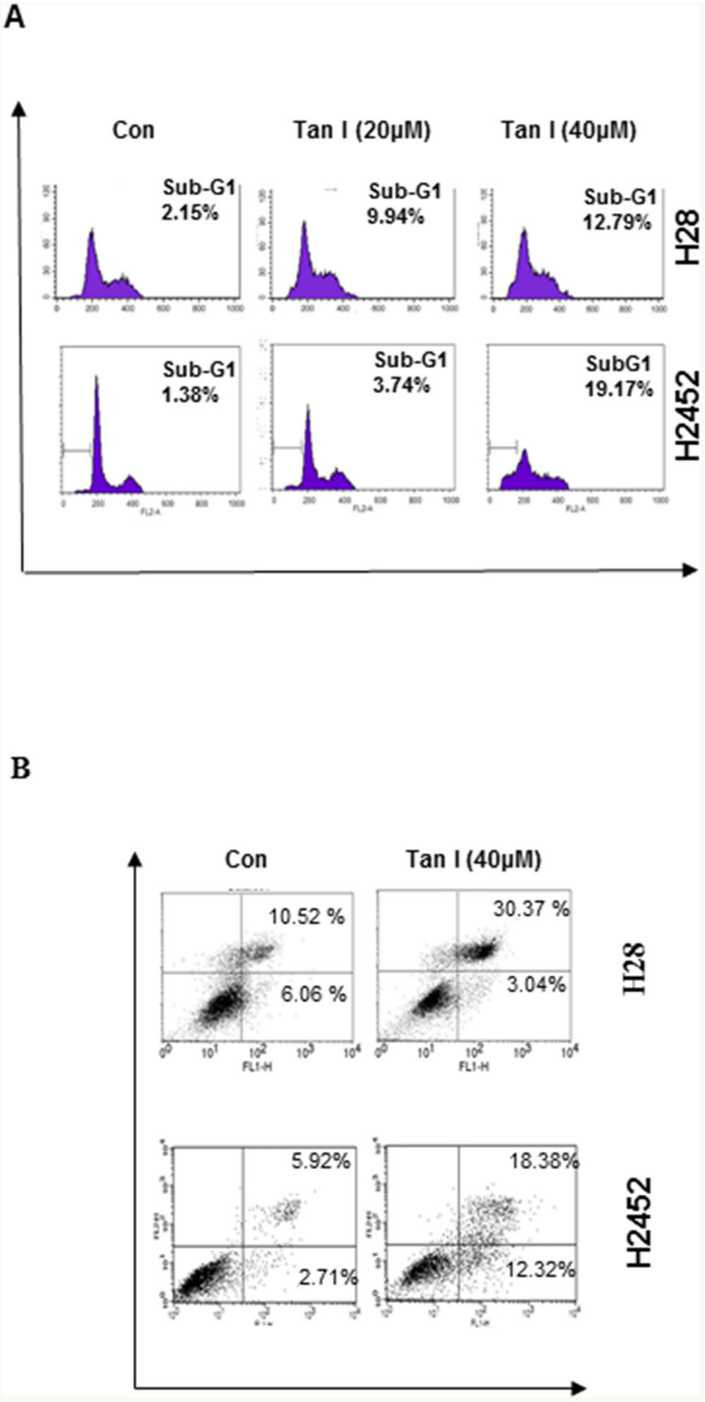

Supplementary Figure 1: Tan I increases sub G1 and apoptotic portion in H28 and H2452 mesothelioma cells by Flow cytometric analysis. A. Effect of Tan I on sub G1 population in H28 and H2452 cells. H28 and H2452 cells were exposed to Tan I (20 and 40 µM) for 24 h, collected, and fixed in 70% ethanol. The cells were then incubated at 37°C with 0.1% ribonuclease (RNase) A in PBS for 30 min and suspended in PBS containing 30µg/mL propidium iodide (PI) for 30 min at room temperature. Sub-G1 population was evaluated from the stained cells by FACSCalibur (Becton Dickinson, Franklin Lakes, NJ, USA) using the Cell Quest program. **B.** Effect of Tan I on apoptotic portion in H28 and H2452 cells by Annexin V and PI doueble staining. H28 and H2452 cells were treated with Tan I (40 µM) for 24 h , then stained with Annexin-V and PI and analyzed by using FACSCalibur flow cytometer.
